# Hyperbranched Polycarbosiloxanes: Synthesis by Piers-Rubinsztajn Reaction and Application as Precursors to Magnetoceramics

**DOI:** 10.3390/polym12030672

**Published:** 2020-03-17

**Authors:** Huayu Zhang, Lei Xue, Jianquan Li, Qingyu Ma

**Affiliations:** 1 Shandong Provincial Key Laboratory of Preparation and Measurement of Building Materials, University of Jinan, Jinan 250022, China; zhy2327212@163.com (H.Z.); mse_lijq@ujn.edu.cn (J.L.); 2 School of Materials Science and Engineering, University of Jinan, Jinan 250022, China; 3 Beijing Institute of Aeronautical Materials, Beijing 100095, China; xuelei282@163.com

**Keywords:** hyperbranched polymers, Piers–Rubinsztajn reaction, polycarbosiloxanes, magnetic ceramics

## Abstract

Silicon-containing hyperbranched polymers (Si-HBPs) have drawn much attention due to their promising applications. However, the construction of Si-HBPs, especially those containing functional aromatic units in the branched backbones by the simple and efficient Piers-Rubinsztajn (P–R) reaction, has been rarely developed. Herein, a series of novel hyperbranched polycarbosiloxanes were prepared by the P–R reactions of methyl-, or phenyl-triethoxylsilane and three Si–H containing aromatic monomers, including 1,4-bis(dimethylsilyl)benzene, 4,4′-bis(dimethylsilyl)-1,1′-biphenyl and 1,1′-bis(dimethylsilyl)ferrocene, using B(C_6_F_5_)_3_ as the catalyst for 0.5 h at room temperature. Their structures were fully characterized by Fourier transform infrared spectroscopy, ^1^H NMR, ^13^C NMR, and ^29^Si NMR. The molecular weights were determined by gel permeation chromatography. The degrees of branching of these polymers were 0.69–0.89, which were calculated based on the quantitative ^29^Si NMR spectroscopy. For applications, the ferrocene-linked Si-HBP can be used as precursors to produce functional ceramics with good magnetizability after pyrolysis at elevated temperature.

## 1. Introduction

Hyperbranched polymers (HBPs), which are defined as highly branched three-dimensional macromolecules, have attracted great attention, due to their unique structures and properties such as abundant functional groups, intramolecular cavities, low viscosity and high solubility, and extensive applications in photoelectric materials, coatings, adhesives, membranes, sensors, catalysis and biomaterials [1,2,3,4,5,6,7]. Among these HBPs, silicon-containing HBPs (Si-HBPs) are one of most important species and have attracted specific interest due to their potentials as silicon carbide ceramic precursors, degradable template molecules, optical materials, modifiers of composites, cell imaging, drug delivery, and gas chromatography [8,9,10,11,12,13,14,15]. The Si-HBPs can be prepared by several strategies, including hydrosilylation reactions from silicon-containing monomers containing Si–H bonds and unsaturated bonds (e.g., vinyl or alkynyl) [16,17], organometallic reaction (e.g., Grignard reaction) [13,18] and polycondensation reaction [19]. However, these strategies have some disadvantages. For example, the most commonly used hydrosilylation reaction requires noble metal-based catalysts, typically Platinum-based Speier’s and Karstedt’s catalysts, and can be affected by moisture and not tolerant for some functional groups. The organometallic reactions are sensitive to moisture and air. To overcome these drawbacks, we and other researchers have introduced click reactions to prepare Si-HBPs [12,20,21]. For example, Wu et al. developed a one-pot thiol-ene approach to prepare polyhedral oligomeric silsesquioxane (POSS)-embedded HBPs with tunable POSS units and terminal vinyl groups from an AB_7_ POSS monomer [12]. We used the step-growth thiol-ene reaction to prepare Si-HBPs from mercaptopropylmethyldiallylsilane (AB_2_) or mercaptopropyltriallylsilane (AB_3_) as the hyperbranched monomers [21]. However, compared to diversified synthetic strategies for conventional HBPs, the strategies for the synthesis of Si-HBPs are still limited. In particular, developing Si-HBPs via simple, rapid and mild synthetic procedures is highly desirable. 

The Piers–Rubinsztajn (P–R) reaction, namely the condensation of alkoxysilanes with hydrosilanes, has rapidly developed since it was found in the 1990s [22,23], because this strategy is mild and provides a possibility for the controlled or precise synthesis of silicones, including small molecules, linear polymers, and crosslinked materials [24,25,26,27,28,29,30,31]. For example, the P–R coupling reactions of various organic tris(dimethylsiloxy)silane and trialkoxysilane compounds can generate a series of cyclic polysiloxanes with cyclotetrasiloxane subunits in a simple one-step synthesis [32]. Matsumoto et al. presented a highly selective sequence-controlled synthesis of linear, branched, and cyclic oligosiloxanes by the iteration of tris(pentafluorophenyl)borane (B(C_6_F_5_)_3_)-catalyzed P–R reaction and hydrosilylation of carbonyl compounds [33]. The P–R reaction has also been applied to synthesize Si-HBPs [34,35,36,37]. For example, Liu et al. reported hyperbranched POSS-based polymers with ultra-high molecular weight by the P–R reaction between octakis(dimethylsiloxy)octa- silsesquioxane with different dialkoxysilanes [34]. However, compared to other synthetic strategies for Si-HBPs, the reports on Si-HBPs, in particular those containing functional aromatic units in the branched backbones prepared by the P–R reaction are still few.

**Scheme 1:**
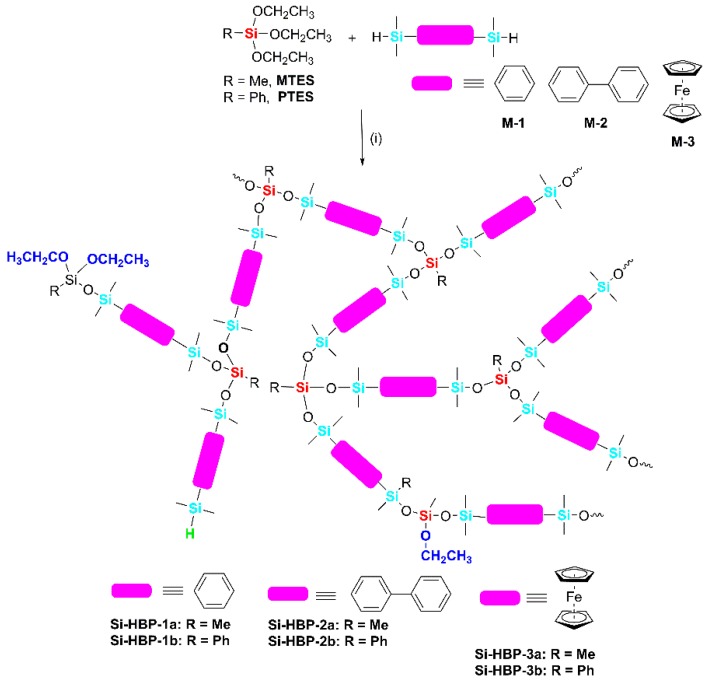
Synthetic routes of hyperbranched polycarbosiloxanes, Si-HBP-1 to Si-HBP-3, by the Piers–Rubinsztajn reaction. (i): B(C_6_F_5_)_3_, toluene, room temperature, 30 min.

Herein, we present a series of novel hyperbranched polycarbosiloxanes, Si-HBP-1 to Si-HBP-3, based on methyl-, or phenyl-triethoxylsilane and three Si–H containing aromatic monomers, including 1,4-bis(dimethylsilyl)benzene (M-1), 4,4′-bis(dimethylsilyl)-1,1′-biphenyl (M-2) and 1,1′-bis(dimethylsilyl) ferrocene (M-3), in the presence of B(C_6_F_5_)_3_ as the catalyst by the P–R reactions (Scheme 1). The obtained Si-HBPs were fully characterized by Fourier transform infrared spectroscopy (FT-IR), ^1^H NMR, ^13^C NMR, ^29^Si NMR and gel permeation chromatography (GPC). Moreover, the pyrolysis of ferrocene-based polycarbosiloxanes at elevated temperatures can afford novel nanostructured ceramics with good magnetizability in a high yield.

## 2. Materials and Methods 

### 2.1. Materials and Characterization

Unless otherwise noted, all reagents were obtained from commercial suppliers and used without further purification. Toluene was dried by distillation from the sodium ketyl of benzophenone. Fourier transform infrared (FT-IR) spectra of the products were recorded on a Bruker Tensor27 spectrophotometer (Ettlingen, Germany) in the frequency range 4000–400 cm^−1^ at a resolution of 4 cm^−1^ with a total of 32 scans. ^1^H NMR and ^13^C NMR spectra were measured on a Bruker AVANCE-300 or 400 NMR spectrometer (Karlsruhe, Germany), with CDCl_3_ as the solvent. Quantitative ^29^Si NMR spectroscopy (Karlsruhe, Germany) was performed using an inverse gated ^1^H-decoupling sequence. Samples were prepared in Cr(acac)_3_/CDCl_3_ solution (0.1 M Cr(acac)_3_). The molecular weights of the polymers and their polydispersity indices (PDI, *M*_w_/*M*_n_) were determined by a Waters 1515 gel permeation chromatography (GPC) system equipped with a refractive index detector (Milford, MA, USA), using monodisperse polystyrene (PS) as calibration standards and THF containing 0.05 M LiBr as the eluent at a flow rate of 1.0 mL min^−1^. Thermogravimetric analysis (TGA) was performed using a TA SDTQ600 (Columbus, OH, USA) with a heating rate of 10 °C/min from room temperature to 1000 °C under N_2_. Differential scanning calorimetry (DSC) was performed on a Netzsch DSC 204 Phoenix (Columbus, OH, USA) and measurements were done under nitrogen atmosphere at a heating rate of 10 °C/min from -160°C to room temperature. X-ray diffraction (XRD) experiments were performed on a Philips PW 2830 X-ray powder diffractometer (Karlsruhe, Germany), with monochromatized Cu Kα radiation (λ = 1.5406 Å). Magnetization curves were performed by using a Lake Shore 7037/9509-P vibrating sample magnetometer at room temperature.

### 2.2. Synthesis of Si-H Containing Aromatic Monomers

1,4-Bis(dimethylsilyl)benzene (M-1), 4,4′-bis(dimethylsilyl)-1,1′-biphenyl (M-2) and 1,1′-bis(dimethylsilyl)ferrocene (M-3) were prepared according to previous reports [17,38]. 

For M-1: IR (KBr pellet cm^−1^): 3047, 2959, 2907, 2123, 1417, 1381, 1250, 1130, 870, 828, 755, 724, 656, 625, 484, 469. ^1^H NMR (400 MHz, CDCl_3_) δ 7.64 (s, 4H), 4.55–4.51 (m, 2H), 0.44 (s, 12H). ^13^C NMR (100 MHz, CDCl_3_): δ 137.6, 132.5, −4.7. ^29^Si NMR (75 MHz, CDCl_3_): δ −17.05.

For M-2: IR (KBr pellet cm^−1^): 3047, 2955, 2123, 1598, 1397, 1253, 1116, 876, 745, 728, 525. ^1^H NMR (400 MHz, CDCl_3_) δ 7.69 (dd, 8H), 4.58–4.55 (m, 2H), 0.45 (d, 12H). ^13^C NMR (100 MHz, CDCl_3_): δ 141.8, 136.4, 134.5, 128.7, 127.0, 126.6, −3.7. ^29^Si NMR (75 MHz, CDCl_3_): δ −17.12.

For M-3: ^1^H NMR (400 MHz, CDCl_3_) δ 7.69 (dd, 8H), 4.58–4.55 (m, 2H), 0.45 (d, 12H). ^13^C NMR (100 MHz, CDCl_3_): δ 73.8, 71.7, 68.3, −3.0. ^29^Si NMR (75 MHz, CDCl_3_): δ −16.85.

### 2.3. Synthesis of Si-H Containing Aromatic Monomers

All the hyperbranched polycarbosiloxanes were prepared from methyl-, or phenyl-triethoxylsilane and Si–H containing aromatic monomers with approximately equimolar amounts of ethoxy groups and Si–H groups. Attention: there was a relatively large amount of bubbles and exotherm during the process.

Si-HBP-1a: A 250 mL flask was charged with 100 mL toluene, M-1 (1.50 g, 7.48 mmol) and B(C_6_F_5_)_3_ (0.5 mol % of Si–H group, 0.0197g, 0.0375 mmol). The mixture was stirred at room temperature for 5 min. Methyltriethoxysilane (MTES, 0.9077g, 4.99 mmol) in toluene (10 mL) was slowly added into the mixture. After a short induction period, gas was vigorously evolved from the solution with heat release. The reaction mixture was stirred at room temperature for 30 min and neutral alumina (~1 g) was added into the mixture. The resulting slurry was stirred for another 20 min, after which alumina was removed by vacuum filtration. The filtrate was distilled under vacuum to remove residual solvent and starting materials, affording the final product as a colorless and viscous liquid (1.90 g, yield: 86%). IR (KBr pellet cm^−1^): 3052, 2959, 1248, 1139, 1041, 819, 772, 669, 612. ^1^H NMR (400 MHz, CDCl_3_) δ 7.72–7.59 (m, −SiC_6_*H*_4_−), 7.48-7.42 (m, −SiC_6_*H*_4_−), 3.85 (d, −SiOC*H*_2_CH_3_), 1.34–1.27 (m, −SiOCH_2_C*H*_3_), 0.48–0.02 (m, −SiC*H*_3_). ^13^C NMR (100 MHz, CDCl_3_): δ 140.6, 140.3, 139.9, 132.5, 132.4, 57.9, 18.3, 0.98, 0.73, 0.64, 0.50, -1.85, −3.52. ^29^Si NMR (75 MHz, CDCl_3_): δ −0.91, −1.23, −1.67, −1.74, −1.95, −2.11, −2.26, −56.67, −57.16, −63.89, −64.34, −64.63. Anal. Calcd for C_16_H_27_O_3_Si_4_: C, 50.61; H, 7.17. Found: C, 50.81; H, 7.95.

Si-HBP-1b: The synthesis and post-treatment procedures of Si-HBP-1b were similar to those of Si-HBP-1a, except that MTES was replaced by phenyltriethoxysilane (PTES, 1.2230 g, 4.99 mmol). The product was afforded as a colorless and viscous liquid (2.1 g, yield: 84%). IR (KBr pellet cm^−1^): 3057, 3006, 2959, 1428, 1377, 1258, 1134, 1041, 814, 772, 720, 694, 674, 612, 555. ^1^H NMR (400 MHz, CDCl_3_) δ 7.69–7.60 (m, −SiC_6_*H*_4_−, −SiC_6_*H*_5_), 7.53-7.28 (m, −SiC_6_*H*_4_−, −SiC_6_*H*_5_), 3.84 (d, −SiOC*H*_2_CH_3_), 1.28–1.20 (m, −SiOCH_2_C*H*_3_), 0.47–0.07 (m, −SiC*H*_3_). ^13^C NMR (100 MHz, CDCl_3_): δ 140.1, 139.9, 134.3, 134.2, 134.0, 132.6, 132.4, 130.1, 129.9, 129.8, 129.7, 129.1, 128.3, 127.9, 127.7, 127.6, 58.6, 58.5, 18.2, 0.96, 0.83, 0.71, 0.67, 0.50, 0.48, 0.42. ^29^Si NMR (75 MHz, CDCl_3_): δ −0.46, −0.51, −0.67, −0.94, −1.26, −70.71, −71.15, −78.04, −78.08. Anal. Calcd for C_21_H_29_O_3_Si_4_: C, 57.09; H, 6.62. Found: C, 57.24; H, 7.70.

Si-HBP-2a: 4,4′-Bis(dimethylsilyl)-1,1′-biphenyl (M-2, 1.50 g, 5.54 mmol), B(C_6_F_5_)_3_ (0.0146 g, 0.0277 mmol) and MTES (0.6726 g, 3.70 mmol) were used to prepare Si-HBP-2a. The synthesis and post-treatment procedures were similar to those of Si-HBP-1a. The product was afforded as a colorless and viscous liquid (1.8 g, yield: 89%). IR (KBr pellet cm^−1^): 3067, 3016, 2959, 2923, 1744, 1594, 1532, 1480, 1434, 1382, 1248, 1123, 1036, 1000, 813, 778, 721, 695, 632, 560. ^1^H NMR (400 MHz, CDCl_3_) δ 7.83–7.73 (m, −SiC_6_*H*_4_−C_6_*H*_4_Si−), 7.64–7.60 (m, −SiC_6_*H*_4_−C_6_*H*_4_Si−), 7.57–7.53 (m, −SiC_6_*H*_4_−C_6_*H*_4_Si−), 3.97–3.89 (m, −SiOC*H*_2_CH_3_), 1.43–1.32 (m, −SiOCH_2_C*H*_3_), 0.58-0.16 (m, −SiC*H*_3_). ^13^C NMR (100 MHz, CDCl_3_): δ 142.1, 141.5, 140.9, 138.5, 138.4, 138.2, 138.1, 138.0, 134.1, 133.9, 133.8, 133.7, 129.1, 128.4, 126.6, 126.5, 126.4, 126.3, 126.1, 58.2, 58.1, 18.5, 18.4, 1.1, 0.9, 0.8, 0.7, −1.8, −3.4, −5.1. ^29^Si NMR (75 MHz, CDCl_3_): δ −0.87, −1.39, −1.42, −1.63, −1.75, −1.93, −48.35, −56.58, −56.89, −63.76, −64.06, −64.11. Anal. Calcd for C_25_H_33_O_3_Si_4_: C, 60.80; H, 6.74. Found: C, 60.99; H, 8.12.

Si-HBP-2b: The synthesis and post-treatment procedures of Si-HBP-2b were similar to those of Si-HBP-2a, except that MTES was replaced by PTES (0.9068 g, 3.70 mmol). The product was afforded as a colorless and viscous liquid (1.91 g, yield: 84%). IR (KBr pellet cm^−1^): 3067, 3021, 2954, 2928, 1744, 1599, 1486, 1428, 1248, 1124, 1036, 996, 824, 777, 721, 700, 632, 555. ^1^H NMR (400 MHz, CDCl_3_) δ 7.75–7.39 (m, −SiC_6_*H*_4_ −C_6_*H*_4_Si−, −SiC_6_*H*_5_), 3.97–3.89 (m, −SiOC*H*_2_CH_3_), 1.35–1.26 (m, −SiOCH_2_C*H*_3_), 0.55–0.09 (m, −SiC*H*_3_). ^13^C NMR (100 MHz, CDCl_3_): δ 142.0, 141.5, 141.0, 138.1, 138.0, 134.6, 134.3, 134.0, 133.8, 133.7, 133.6, 130.1, 130.0, 128.0, 127.9, 127.8, 126.6, 126.5, 126.3, 126.1, 58.7, 58.6, 18.4, 18.3, 1.1, 0.80, 0.65. ^29^Si NMR (75 MHz, CDCl_3_): δ 0.21, −0.22, −0.27, −0.44, −0.64, −0.95, −63.93, −70.95, −77.72, −77.85. Anal. Calcd for C_30_H_35_O_3_Si_4_: C, 64.81; H, 6.35. Found: C, 64.34; H, 7.15.

Si-HBP-3a: 1,1’-Bis(dimethylsilyl)ferrocene (M-3, 2.00 g, 6.62 mmol), B(C_6_F_5_)_3_ (0.0174 g, 0.0331 mmol) and MTES (0.8023 g, 4.41 mmol) were used to prepare Si-HBP-3a. The synthesis and post-treatment procedures were similar to those of Si-HBP-1a. The product was afforded as a colorless and viscous liquid (1.90 g, yield: 72%). IR (KBr pellet cm^−1^): 3088, 2959, 2897, 1418, 1356, 1248, 1165, 1036, 959, 901, 813, 773, 689, 669, 622. ^1^H NMR (400 MHz, CDCl_3_) δ 4.39–4.15 (m, −C_5_*H*_4_FeC_5_*H*_4_−), 3.89–3.74 (m, −SiOC*H*_2_CH_3_), 1.29–1.19 (m, −SiOCH_2_C*H*_3_), 0.44–0.29 (m, −SiC*H*_3_). ^13^C NMR (100 MHz, CDCl_3_): δ 73.7, 73.6, 73.4, 73.2, 73.1, 73.0, 72.9, 71.7, 71.2, 70.9, 70.8, 70.7, 68.4, 68.0, 58.0, 18.4, 18.3, 1.9, 1.7, 1.4, 1.3, 1.2, 0.2, −2.0, −3.4. ^29^Si NMR (75 MHz, CDCl_3_): δ 1.53, 1.26, 0.87, 0.78, 0.58, 0.38, 0.29, 0.23, 0.16, −0.03, −0.53, −49.84, −57.42, −57.48, −58.70, −64.99, −66.22, −66.27. Anal. Calcd for C_22_H_33_O_3_Si_4_Fe_1.5_: C, 48.79; H, 6.14. Found: C, 49.81; H, 7.51.

Si-HBP-3b: The synthesis and post-treatment procedures of Si-HBP-3b were similar to those of Si-HBP-3a, except that MTES was replaced by PTES (1.0602 g, 4.41 mmol). The product was afforded as a colorless and viscous liquid (2.14 g, yield: 74%). IR (KBr pellet cm^−1^): 3093, 3073, 2959, 2897, 1651, 1591, 1428, 1367, 1300, 1253, 1168, 1129, 1036, 959, 897, 808, 778, 736, 721, 700, 674, 632, 566. ^1^H NMR (400 MHz, CDCl_3_) δ 7.76-7.29 (m, −SiC_6_*H*_5_), 4.40–4.05 (m, −C_5_*H*_4_FeC_5_*H*_4_−) 3.82–3.73 (m, −SiOC*H*_2_CH_3_), 1.27–1.21 (m, −SiOCH_2_C*H*_3_), 0.49–0.01 (m, −SiC*H*_3_). ^13^C NMR (100 MHz, CDCl_3_): δ 134.6, 134.4, 134.1, 130.1, 129.8, 129.5, 127.8, 127.7, 127.6, 127.4, 73.6, 73.5, 73.4, 73.3, 73.2, 72.1, 71.3, 71.2, 70.9, 70.8, 68.4, 58.6, 58.5, 58.4, 18.4, 2.1, 1.8, 1.7, 1.4, 1.2, 0.2. ^29^Si NMR (75 MHz, CDCl_3_): δ 2.67, 2.48, 2.45, 2.08, 1.94, 1.81, 1.78, 1.48, 1.29, 1.13, 0.95, 0.82, 0.03, −64.40, −71.49, −72.02, −78.68, −79.84. Anal. Calcd for C_27_H_35_O_3_Si_4_Fe_1.5_: C, 53.72; H, 5.84. Found: C, 54.63; H, 7.52.

### 2.4. Synthesis of Magnetic Ceramics from Si-HBP-3a

Si-HBP-3a was pyrolyzed at 900 °C for 2 h in a tube furnace with the heating rate of 10 °C/min from temperature to 600 °C, and 5 °C/min from 600 to 900 °C under the flowing nitrogen and then cooled to temperature. The product was obtained as a black powder.

## 3. Results and Discussion

### 3.1. Synthesis

The Si-H containing monomers, M-1 to M-3, were synthesized following the literature procedures [17,38]. Their structures were characterized by ^1^H NMR, ^13^C NMR, and ^29^Si NMR and the satisfactory analysis data were obtained. 

The synthetic routes of silicon-containing HBPs, Si-HBP-1 to Si-HBP-3, were shown in Scheme 1. All the polymers were synthesized by the P–R polymerization reactions of trifunctional ethoxy monomer (MTES and PTES) and bifunctional Si-H monomers (M-1 to M-3). The molar feed ratio of ethoxy groups and Si–H groups was set at approximately 1:1. It is known that controlling the gel time is crucial for the preparation of hyperbranched polymers. Taking the P-R reaction of M-1 and MTES as an example (Si-HBP-1a), different amounts of catalysts, including 0.2, 0.5, 0.8 and 1.0 mol % (calculation based on the amount of Si-H groups), were used to evaluate the reaction rate, while keeping the concentration of reaction mixture constant. Table 1 shows the gel time variation by changing the amount of catalyst. It was found that when the amount is 0.2 mol %, the reaction was very slow and the gel time is ca. 5 h. When the amounts are 0.8 and 1.1 mol %, the reactions were difficult to control and the solutions rapidly became gel. Thus, 0.5 mol % was chosen as the catalyst amount for the reaction. In addition, it was found that after 30 min, the gelation also occurred and the reaction time was selected to be 30 min. All the products were afforded as colorless and viscous liquids and soluble in aliphatic and aromatic solvents.

### 3.2. Characterization

**Figure 1:**
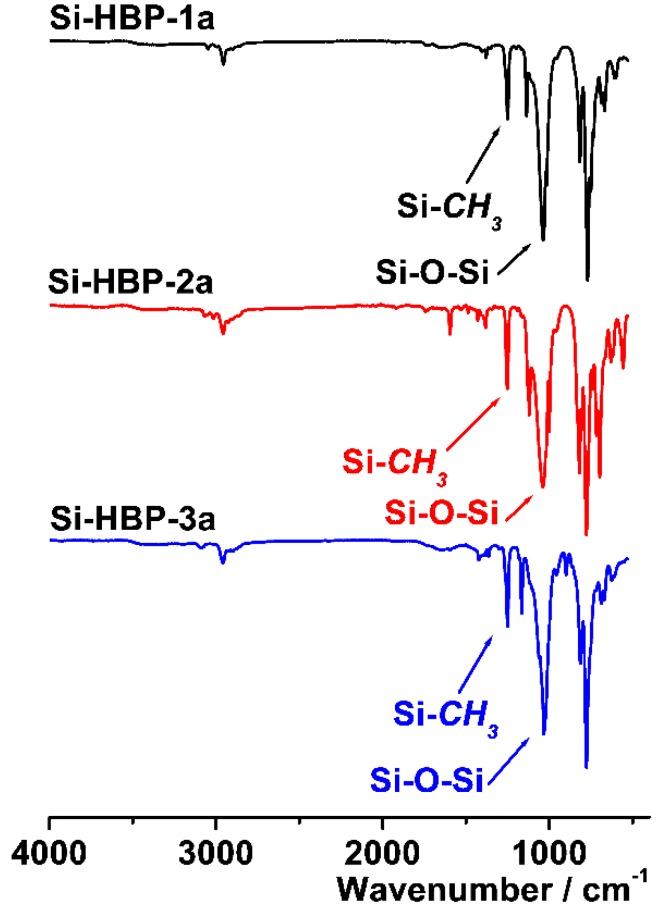
Fourier transform infrared (FT-IR) spectroscopy of Si-HBP-1a, Si-HBP-2a and Si-HBP-3a.

The structures of all the polymers were fully determined by FT-IR, ^1^H NMR, ^13^C NMR, ^29^Si NMR, and elemental analysis. The FT-IR spectra of these polymers were shown in Figure 1 and Figure S1 (Supplementary Materials). The absorption peaks, with moderate intensity ranging from 1650 to 1400 cm^−1^, are associated with C=C stretching vibrations from phenyl or cyclopentadienyl (Cp) units in the networks. The peaks at ca. 1265 cm^−1^ are attributed to the deformation vibrations of methyl groups linked to silicon atoms. Compared with the monomers (taking M-1 as an example), the strong absorption band at 2123 cm^−1^ attributed to the Si–H stretching vibration disappears in the spectrum of Si-HBP-1a, while a new and strong peak at ca. 1035 cm^−1^ undoubtedly assigned to the characteristic Si–O–Si stretching vibration is observed (Figure S2 in Supplementary Materials). Similar results are also obtained in other polymers. This finding reveals that most of the Si–H groups have been consumed after the polymerization reaction, thereby indicating that the target polymers have been successfully synthesized.

**Figure 2:**
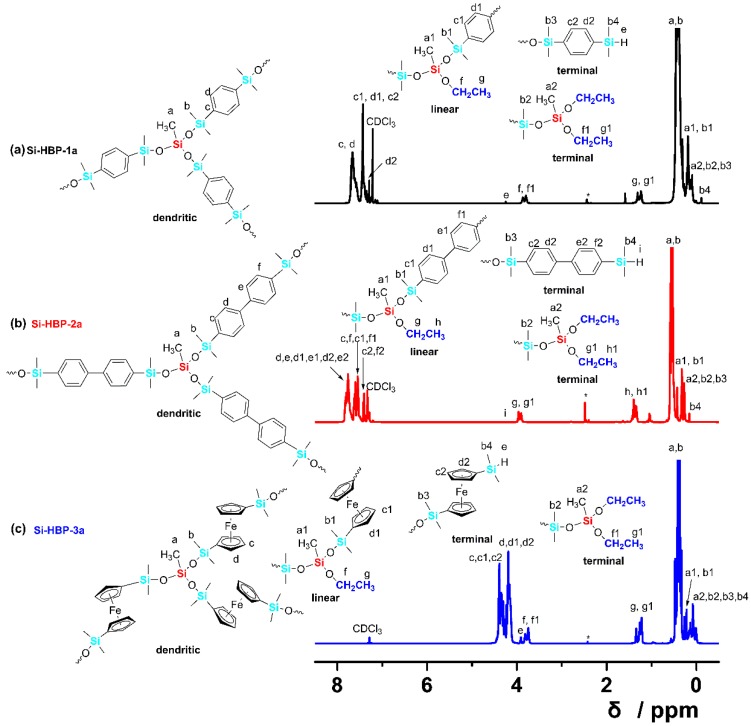
^1^H NMR spectra of Si-HBP-1a (**a**), Si-HBP-2a (**b**) and Si-HBP-3a (**c**). The asterisk denotes the residual toluene in the product.

**Figure 3:**
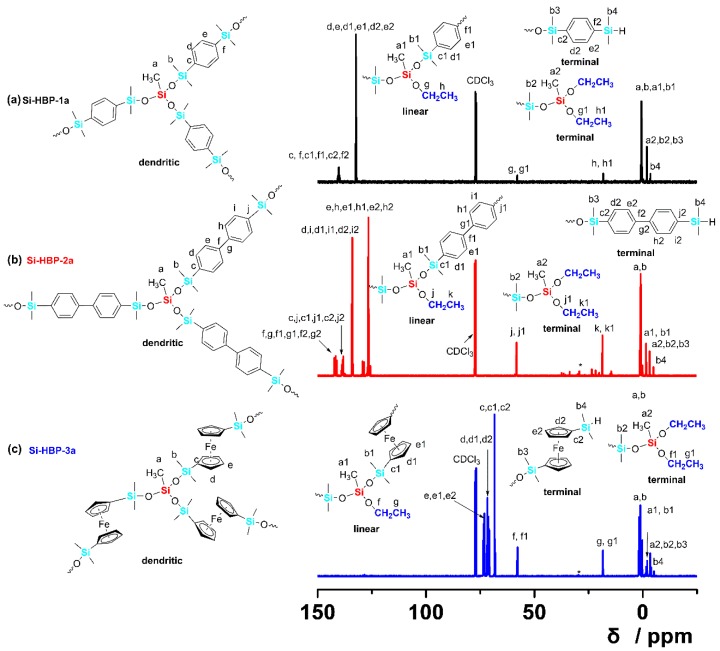
^13^C NMR spectra of Si-HBP-1a (**a**), Si-HBP-2a (**b**) and Si-HBP-3a (**c**). The asterisk denotes the residual toluene in the product.

Figure 2 and Figure S3 (Supplementary Materials) show the ^1^H NMR spectra of these polymers in CDCl_3_ and the assignments of the resonance signals. Similar to other hyperbranched polymers, these polymers also possess dendritic (D), linear (L) and terminal (T) units [5]. Compared to the starting monomers (M-1 to M-3), the characteristic signals of proton on Si–H bond (4.51–4.58 ppm, see the experimental section) in these polymers approximately disappeared. Meanwhile, the signals, which are assigned to the protons in the ethoxy groups, also nearly disappeared and weak peaks at ca. 3.9–3.8 ppm (H_f_ and H_f1_ in Figure 2a, H_g_ and H_g1_ in Figure 2b, H_f_ and H_f1_ in Figure 2c) and 1.4–1.3 ppm (H_g_ and H_g1_ in Figure 2a, H_h_ and H_h1_ in Figure 2b, H_g_and H_g1_ in Figure 2c) were observed for the protons associated with the secondary carbon –SiOCH_2_CH_3_ and the primary carbon –SiOCH_2_CH_3_. The almost disappearance of Si–H bonds and –OCH_2_CH_3_ groups suggested the succesful reactions from the starting materials and both of them have occupied the terminal positions in the networks. For the polymers based on methyltriethoxysilane and M-1 to M-3, the peaks at 0–0.5 ppm are obviously attributed to the protons of Si–CH_3_ groups. The signals from 7.2 ppm to 7.8 ppm are assigned to the aromatic protons from the phenyl groups for all the polymers except Si-HBP-3a. For Si-HBP-3a and Si-HBP-3b, the signals at around 4.15 ppm and 4.35 ppm corresponded to the protons on the Cp rings (Figure 2c and Figure S3c in Supplementary Materials). Moreover, for Si-HBP-3b, the multiple peaks in the region of 7.2–7.8 ppm arising from the phenyl units (Figure 3c) proved that it was synthesized from PTES, which is different from that of Si-HBP-3a.

Further evidence can be found in ^13^C NMR spectra of these polymers in CDCl_3_ with the assignments of the resonance signals (Figure 3 and Figure S4 in Supplementary Materials). Similar to the results in ^1^H NMR spectra, the characteristic signals of –SiOCH_2_CH_3_ carbons are found at ca. 58 ppm (C_g_ and C_g1_ in Figure 3a, C_j_ and C_j1_ in Figure 3b, C_f_ and C_f1_ in Figure 3c) and 18 ppm (C_h_ and C_h1_ in Figure 3a, C_k_ and C_k1_ in Figure 3b, C_g_ and C_g1_ in Figure 3c) from the secondary carbon –SiOCH_2_CH_3_ and the primary carbon –SiOCH_2_CH_3_. As expected, the multiple peaks from −5 ppm to 3 ppm are assocated with the carbons from the Si−CH_3_ units depending on the chemical enviroments. The signals in the region of 142–126 ppm are assigned to sp^2^ phenyl carbons atoms from the M-1 to M-3, or PTES for all the polymers except Si-HBP-3a. For the ferrocene-linked polymers (Si-HBP-3a and Si-HBP-3b), the characteristic signals for the carbons on the Cp rings are observed at ca. 68 ppm and 73 ppm (Figure 3c and Figure S4c in Supplementary Materials). For Si-HBP-3b, the introduction of phenyl groups in the network can also be proven by the multiple peaks in the region of 135–127 ppm (Figure S4c).

**Figure 4:**
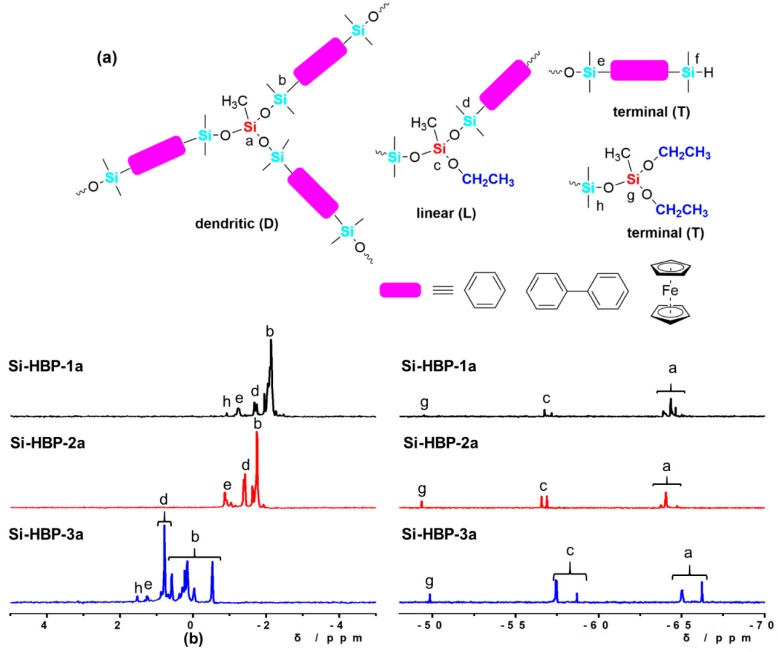
(**a**) The possible Si chemical environments for Si-HBPs; (**b**–**c**) ^29^Si NMR spectra of Si-HBP-1a, Si-HBP-2a and Si-HBP-3a in the region of 5–−5 ppm (**b**) and in the region of −48–−70 ppm (**c**).

For silicon-containing hyperbranched polymers, ^29^Si NMR is an important technique to identify the Si chemical environments in the structures. Figure 4a shows the possible Si chemical structures and Figure 4b,c and Figures S5–S7 (Supplementary Materials) show the ^29^Si NMR spectra of these polymers. For Si-HBP-1a, the silicon atoms linked to three oxygen atoms in the dendritic (Si_a_), linear (Si_c_) and terminal (Si_g_) units are found at ca. −64, −57, −49 ppm (Figure 4c). The signals in the region of −0.9 to −2.3 ppm are assigned to silicon atoms linked to two methyl groups (Si_b_, Si_d_, Si_e_, and Si_h_) (Figure 4b). However, The Si–H was not detected because of its low amount and weak signals. The results of ^29^Si NMR spectra for Si-HBP-2a are similar to those for Si-HBP-1a (Figure 4b,c). For Si-HBP-3a, the introduction of ferrocene leads the signals of Si atoms in Si-CH_3_ groups to move to the low field in the region of 0.01–0.48 ppm (Figure 4b,c). Similar results are also found in other polymers. In addition, there is a small difference between the calculated and theoretical contents of C and H contents in the elemental analysis. This finding may be due to the presence of terminal units, because the theoretical contents were calculated based on the hypothetically complete reaction between Si-H groups and ethoxy groups.

Moreover, based on the quantitative ^29^Si NMR spectroscopy, the degrees of branching (DBs) of these polymers can be determined and calculated according to Equations (1) and (2), based on Frey’s and Fréchet’s definition [39,40]:

(1) $$\mathrm{DB}=\frac{2D}{2D+L}$$

(2) $$\mathrm{DB}=\frac{D+T}{D+T+L}$$

where *D*, *L*, *T* represent the fraction of dendritic, linear, terminal units in hyperbranched polymers. After calculation, the DBs of these polymers were in the range of 0.69 (Si-HBP-3) to 0.89 (Si-HBP-1) and 0.55 (Si-HBP-3a) to 0.88 (Si-HBP-1b) for DB_Frey_ and DB_Fréchet_ (Table 2). The values are similar to other silicon-containing hyperbranched polymers, such as hyperbranched ferrocene-containing poly(boro)carbosilanes (DB_Frey_: 0.50–0.77; DB_Fréchet_: 0.47–0.79) [17], thioether-containing Si-HBP (DB: 0.6) [21]. These results demonstrate that these polymers have highly branched architectures.

The relative molecular weights of these polymers were determined by GPC analysis with polystyrene standards for calibration (Figures S8–S13 in Supplementary Materials). The results are summarized in Table 2. The average molecular weights of these polymers were in the range of 1850 g mol^−1^ (Si-HBP-3b) to 4300 g mol^−1^ (Si-HBP-1a). These values are moderate compared to other silicon-containing hyperbranched polymers [21]. The polydispersity indexes (PDIs) of the resultant polymers were broad and from 2.16 (Si-HBP-3b) to 4.21 (Si-HBP-1a). This phenomenon could be assigned to the features of GPC analysis, which do not always lead to very accurate molecular weight values for hyperbranched polymers. It is interesting that the phenyltriethoxysilane derived products (Si-HBP-1b, 2b and 3b) possess lower molecular weight than methyltriethoxysilane derived products (Si-HBP-1a, 2a and 3a). This finding can be attributed to the relatively lower reaction activity, due to the higher steric hindrance of phenyltriethoxysilane than that of methyltriethoxysilane. The different molecular weight among them may be due to the different cyclization product in the final network, because the intramolecular cyclization reaction is generally unavoidable in the synthesis of hyperbranched polymers.

In addition, the thermal stabilities of the polymers were evaluated by thermogravimetric analysis (TGA) under N_2_ at 10 °C/min. All the polymers exhibit high thermal stability with a high decomposition temperature with T_d_ (5% mass loss) of approximately 350°C (except Si-HBP-1a) and the char yields of >35% at 1000 °C (Figure S14 in Supplementary Materials). The DSC measurements reveal that there is no obvious glass transition temperature (*T*_g_) for any polymer.

### 3.3. Magnetoceramic from Ferrocene-Based Hyperbranched Polymer Si-HBP-3a

Si-containing hyperbranched polymers are generally good precursors for constructing nanostructured Si-containing ceramic materials. In particular, the introduction of ferrocene in the ceramics could impart them magnetism [41,42,43]. Thus, Si-HBP-3a was pyrolyzed in a tube furnace at 900°C for 2 h under a steam of nitrogen and the ceramic product was afforded as a black powder with the yield of 45.6%, consistent with the TGA result. It is interesting that the ceramic can be easily attracted by a magnet, which motivated us to explore its structure and the magnetic property.

**Figure 5:**
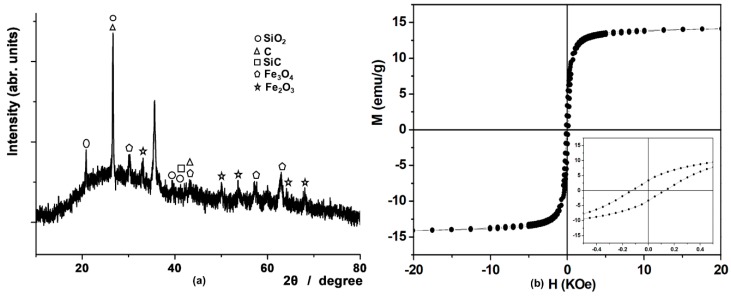
(**a**) XRD pattern of the ceramic pyrolyzed from Si-HBP-3a; (**b**) Plot of magnetization (M) versus applied magnetic field (H) at 300 K for the magnetoceramic.

To gain the chemical composition of the formed ceramic, powder X-ray diffraction (XRD) was carried out (Figure 5a). As expected, the ceramic displays many Bragg reflection peaks, revealing that it possesses different crystalline species. Based on the databases of JCPDS-International Centre for Diffraction Data (ICDD), the serial peaks at 2θ angles, including 20.8°, 26.6°, 30.1°, 33.1°, 35.6°, and 62.6°, etc., are associated with the reflections of SiO_2_, SiC, C, Fe_3_O_4_, and Fe_2_O_3_ (ICDD data file 46-1045, 29-1129, 26-1076, 89-0691 and 33-0664). These results reveal that the hyperbranched polymer has been successfully pyrolyzed and transformed into a ceramic.

The magnetic property of the resultant ceramic was investigated by using a vibrating sample magnetometer (VSM). The magnetization curve of the ceramic was shown in Figure 5b. As the strength of the externally applied magnetic field increases, the magnetization of the ceramic sharply increases and reaches the saturated magnetization of 14.6 emu g^−1^. This value was comparable to some Fe-containing ceramics, such as Fe_3_Si nanocrystal enriched ceramic (C1) [43] and ceramics from hyperbranched poly(ferrocenylene)s, containing group 14 and 15 elements [44]. In addition, the magnetization plot contains hysteresis, as can be seen in the inset of Figure 5b. The coercivity is found to be 0.12 kOe. These results reveal that the ferrocene-linked hyperbranched polycarbosiloxane is a promising precursor for magnetic ceramics.

## 4. Conclusions

In summary, we have prepared a series of novel hyperbranched polycarbosiloxanes by the Piers–Rubinsztajn (P–R) reactions. The polymerization reactions of methyl-, or phenyl- triethoxylsilane and three Si-H containing aromatic monomers were carried out at room temperature for 0.5 h, using B(C_6_F_5_)_3_ as the catalyst. The structures were proved by FT-IR, ^1^H NMR, ^13^C NMR, and ^29^Si NMR. The molecular weights are in the range of 1850 to 4300 g mol^−1^, which are moderate compared to other silicon-containing hyperbranched polymers. The degrees of branching of these polymers were in the range of 0.69–0.89, which were determined by the quantitative ^29^Si NMR. These polymers can be utilized as precursors for the construction of ceramics. Pyrolysis of a ferrocene-linked Si-HBP at elevated temperature resulted in a ceramic with the yield of 45.6%. The resultant ceramic exhibited a good magnetizability, with the saturation magnetization of 14.6 emu g^−1^, which are comparable to those from linear and hyperbranched ferrocene-containing polymers. This work represents a typical example of Si-HBPs prepared by the P–R reactions. The pyrolysis transformation from ferrocene-linked Si-HBP into magnetoceramics imparts them as promising candidates for high-technology applications. More examples could be developed according to this work.

## Figures and Tables

**Table 1:** The gel time versus catalyst amount for the preparation of Si-HBP-1a.

Entry	The Mount of Catalyst (mol %)	Gel Time
1	0.2	ca. 5 h
2	0.5	32 min
3	0.8	< 30 s
4	1.1	< 20 s

**Table 2:** GPC data of Si-HBP-1 to Si-HBP-3.

Samples	*M*_n_ (g mol^−1^) ^a^	*M*_w_ (g mol^−1^) ^a^	PDI ^a^	DB ^b^	DB ^c^
Si-HBP-1a	4300	18100	4.21	0.89	0.81
Si-HBP-1b	3600	14300	3.97	0.89	0.88
Si-HBP-2a	3000	11800	3.93	0.77	0.65
Si-HBP-2b	2800	10100	3.61	0.84	0.83
Si-HBP-3a	1950	4700	2.41	0.69	0.55
Si-HBP-3b	1850	4000	2.16	0.69	0.61

^a^ The molecular weights and the polydispersity index (PDI) were determined by gel permeation chromatography (GPC) analysis, with polystyrene standards for calibration. ^b^ DB_Frey_ was determined by ^29^Si NMR; ^c^ DB_Fréchet_ was determined by ^29^Si NMR.

## Supplementary Materials

The following are available online at https://www.mdpi.com/2073-4360/12/3/672/s1, Figure S1: FT-IR spectroscopy of Si-HBP-1b, Si-HBP-2b and Si-HBP-3b, Figure S2: FT-IR spectroscopy of M-1 and Si-HBP-1a, Figure S3: ^1^H NMR spectra of Si-HBP-1b (a), Si-HBP-2b (b) and Si-HBP-3b (c), Figure S4: ^13^C NMR spectra of Si-HBP-1b (a), Si-HBP-2b (b) and Si-HBP-3b (c), Figure S5: ^29^Si NMR spectra of Si-HBP-1a, Si-HBP-2a and Si-HBP-3a, Figure S6: ^29^Si NMR spectra of Si-HBP-1b, Si-HBP-2b and Si-HBP-3b, Figure S7: (a) The possible Si chemical environments for Si-HBPs; (b-c) ^29^Si NMR spectra of Si-HBP-1b, Si-HBP-2b and Si-HBP-3b in the region of 5−-5 ppm and in the region of -60−-85 ppm, Figure S8: GPC curve of Si-HBP-1a, Figure S9: GPC curve of Si-HBP-1b, Figure S10: GPC curve of Si-HBP-2a, Figure S11: GPC curve of Si-HBP-2b, Figure S12: GPC curve of Si-HBP-3a, Figure S13: GPC curve of Si-HBP-3b. Figure S14: TGA curves of hyperbranched polycarbosiloxanes, Si-HBP-1~Si-HBP-3 under the atmosphere of nitrogen from temperature to 1000 °C.
