# Supplementary material for: Hyperbranched Polycarbosiloxanes: Synthesis by Piers-Rubinsztajn Reaction and Application as Precursors to Magnetoceramics
Source: Polymers (Basel). 2020 Mar 17;12(3):672. doi: 10.3390/polym12030672 (PMC7183318; doi:10.3390/polym12030672)
Supplement: Supplementary file 1 [file polymers-12-00672-s001.pdf]

Supporting Information

Hyperbranched Polycarbosiloxanes: Synthesis by Piers-Rubinsztajn Reaction and Application as Precursors to Magnetoceramics

Huayu Zhang [1,2], Lei Xue [3], Jianquan Li [1,2], and Qingyu Ma [1,2,*]

[1] Shandong Provincial Key Laboratory of Preparation and Measurement of Building Materials, University of Jinan, Jinan 250022, China; zhy2327212@163.com (H.Z.); mse_lijq@ujn.edu.cn (J.L.)
[2] School of Materials Science and Engineering, University of Jinan, Jinan 250022, China
[3] Beijing Institute of Aeronautical Materials, Beijing 100095, China; xuelei282@163.com (L.X.)
* Correspondence: mse_maqy@ujn.edu.cn (Q.M.)

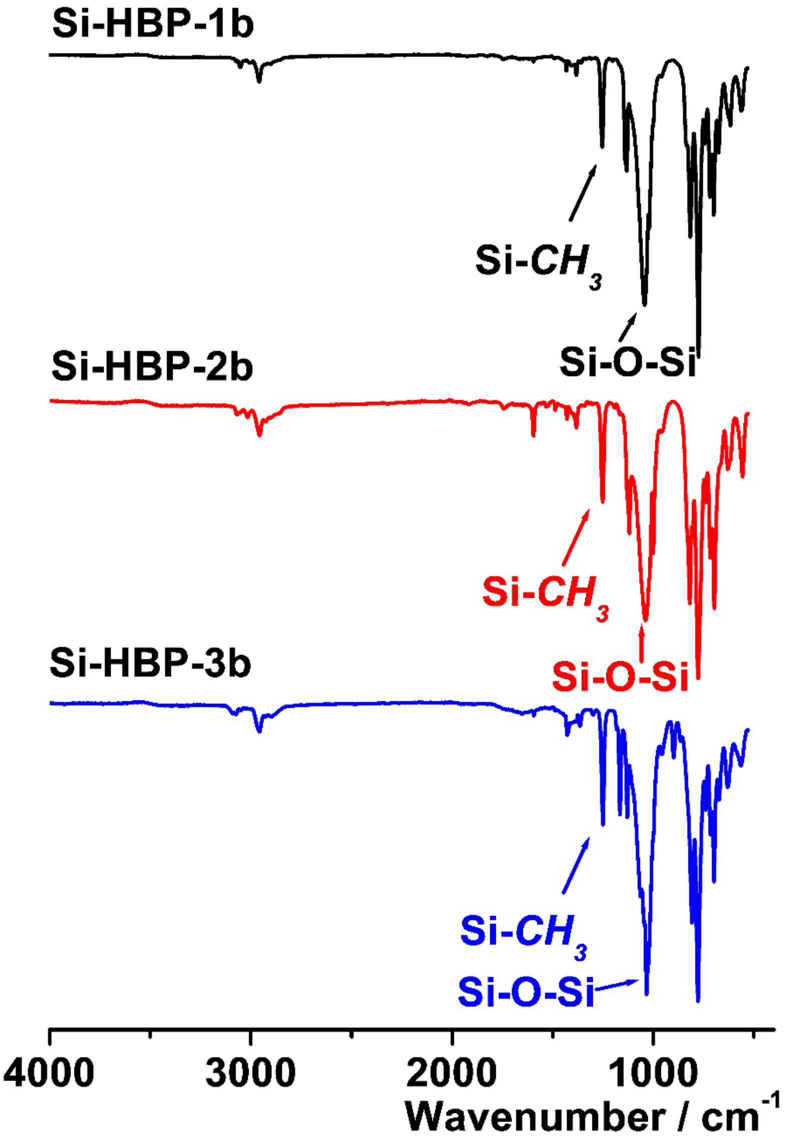

Figure S1. FT-IR spectroscopy of Si-HBP-1b, Si-HBP-2b and Si-HBP-3b.

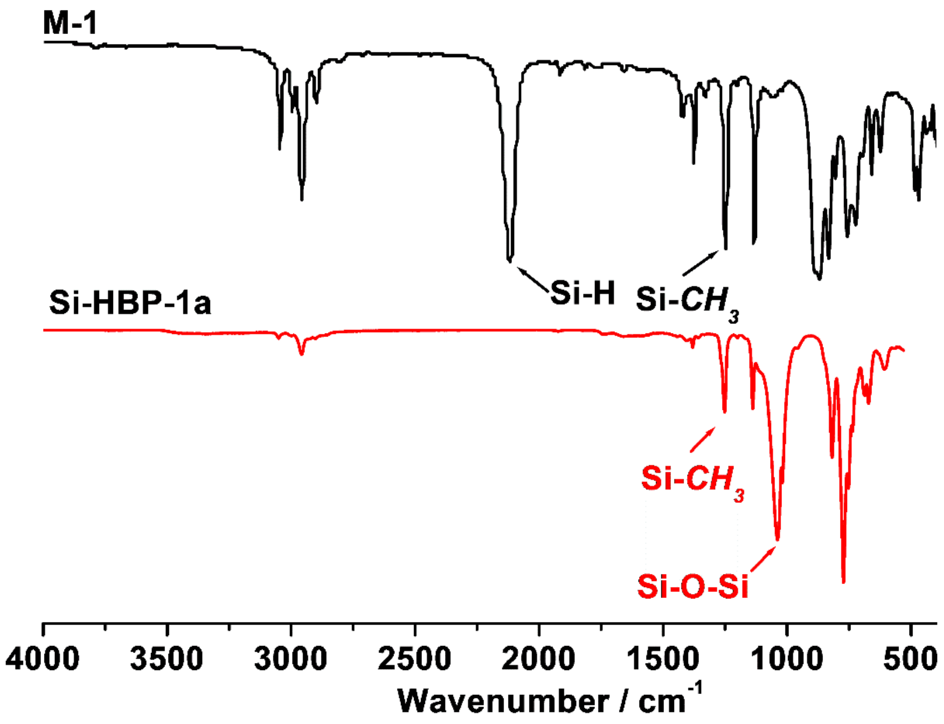

Figure S2. FT-IR spectroscopy of M-1 and Si-HBP-1a.

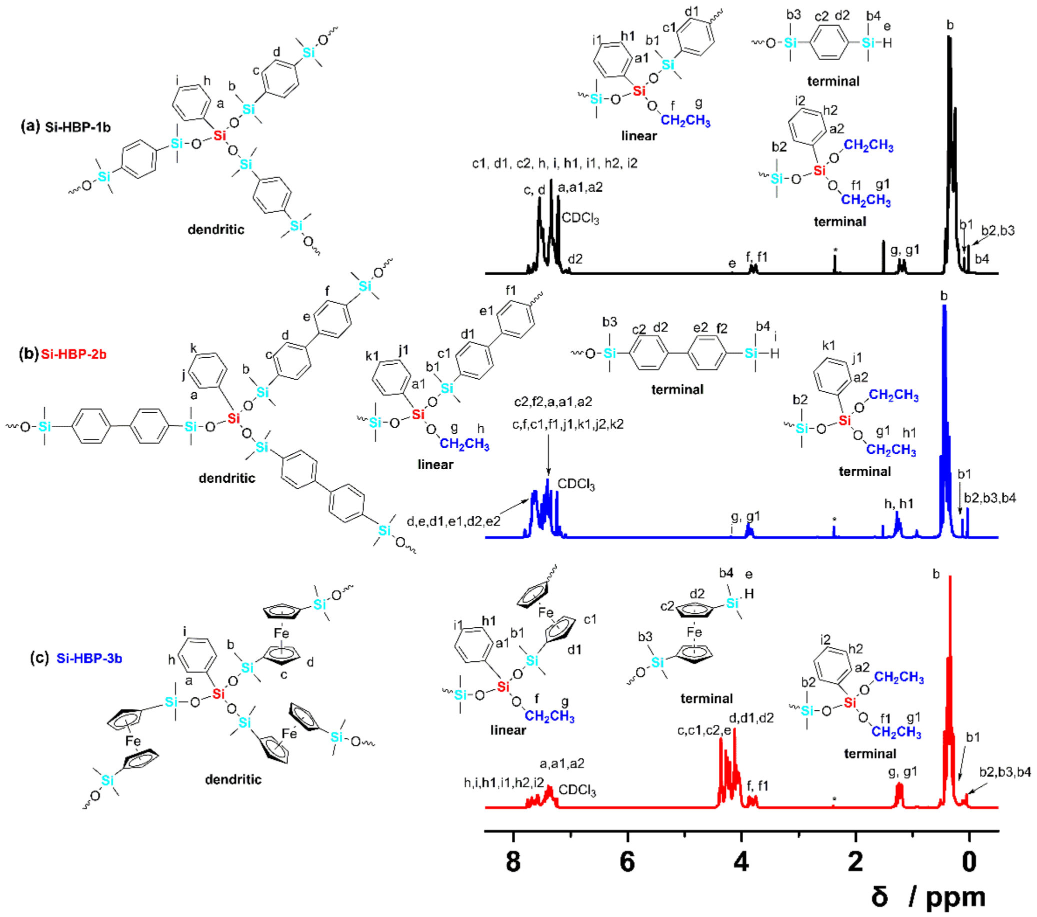

Figure S3. ^{1}H NMR spectra of Si-HBP-1b (a), Si-HBP-2b (b) and Si-HBP-3b (c).

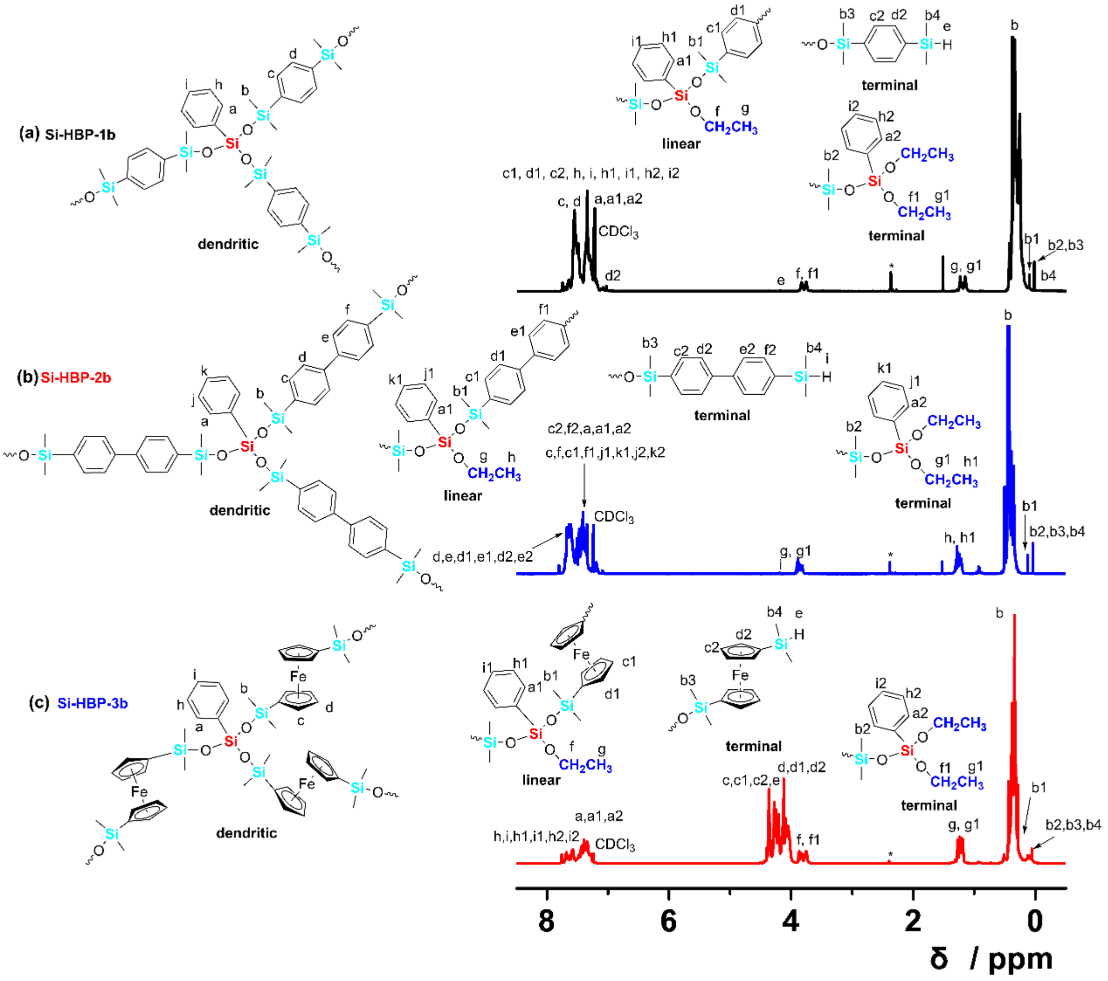

Figure S4. ^{13}C NMR spectra of Si-HBP-1b (a), Si-HBP-2b (b) and Si-HBP-3b (c).

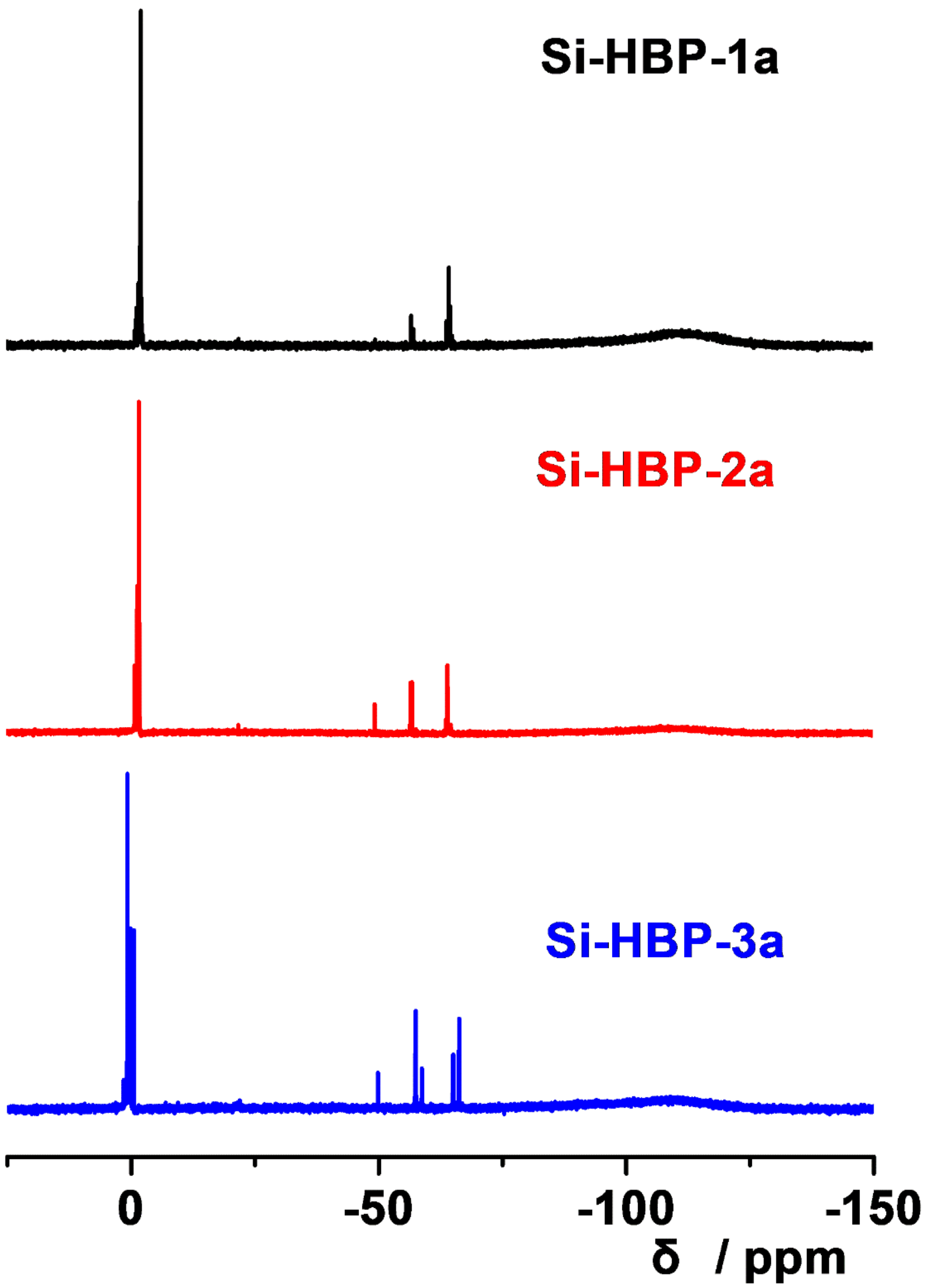

Figure S5. ^{29}Si NMR spectra of Si-HBP-1a, Si-HBP-2a and Si-HBP-3a.

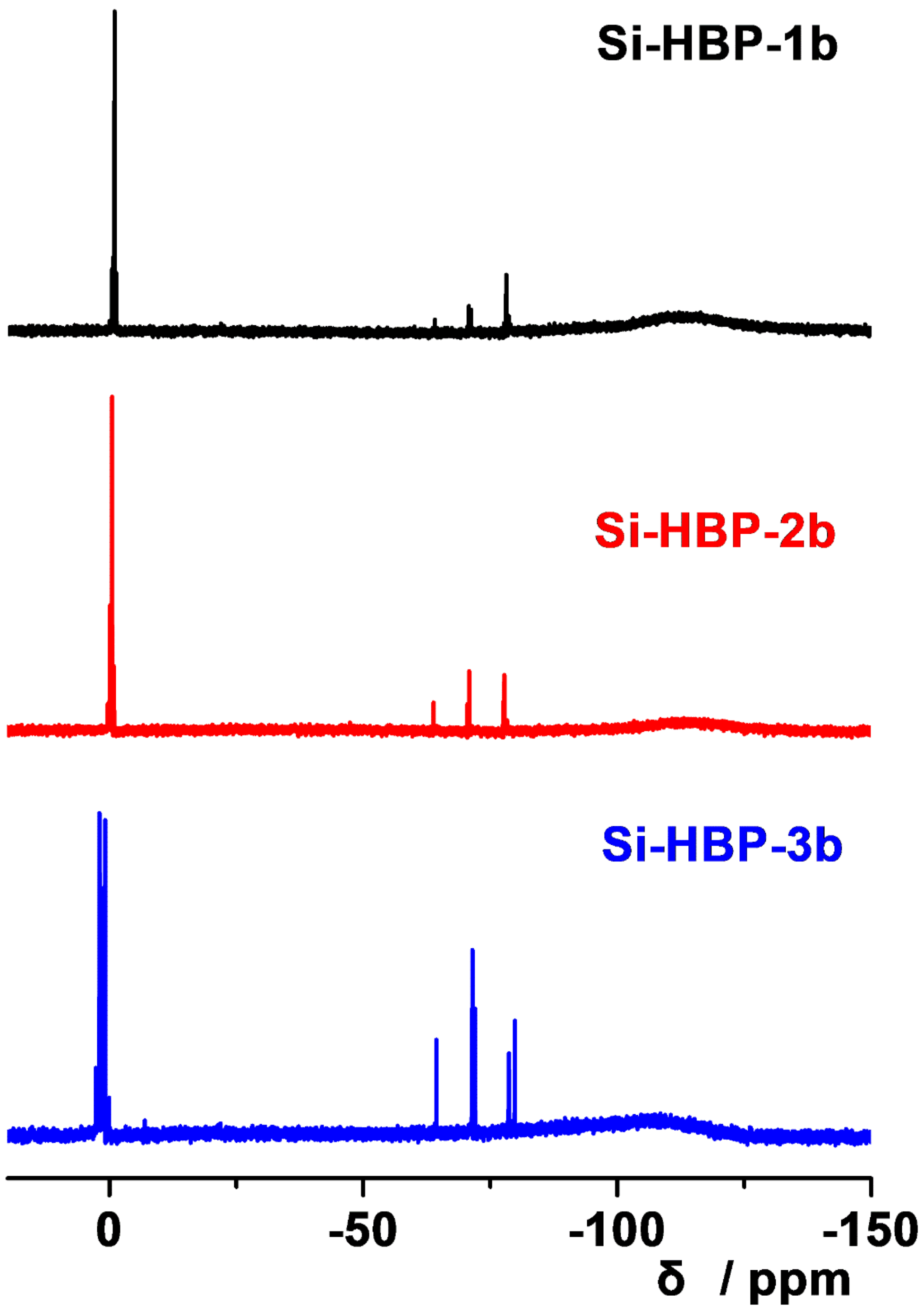

Figure S6. ^{29}Si NMR spectra of Si-HBP-1b, Si-HBP-2b and Si-HBP-3b.

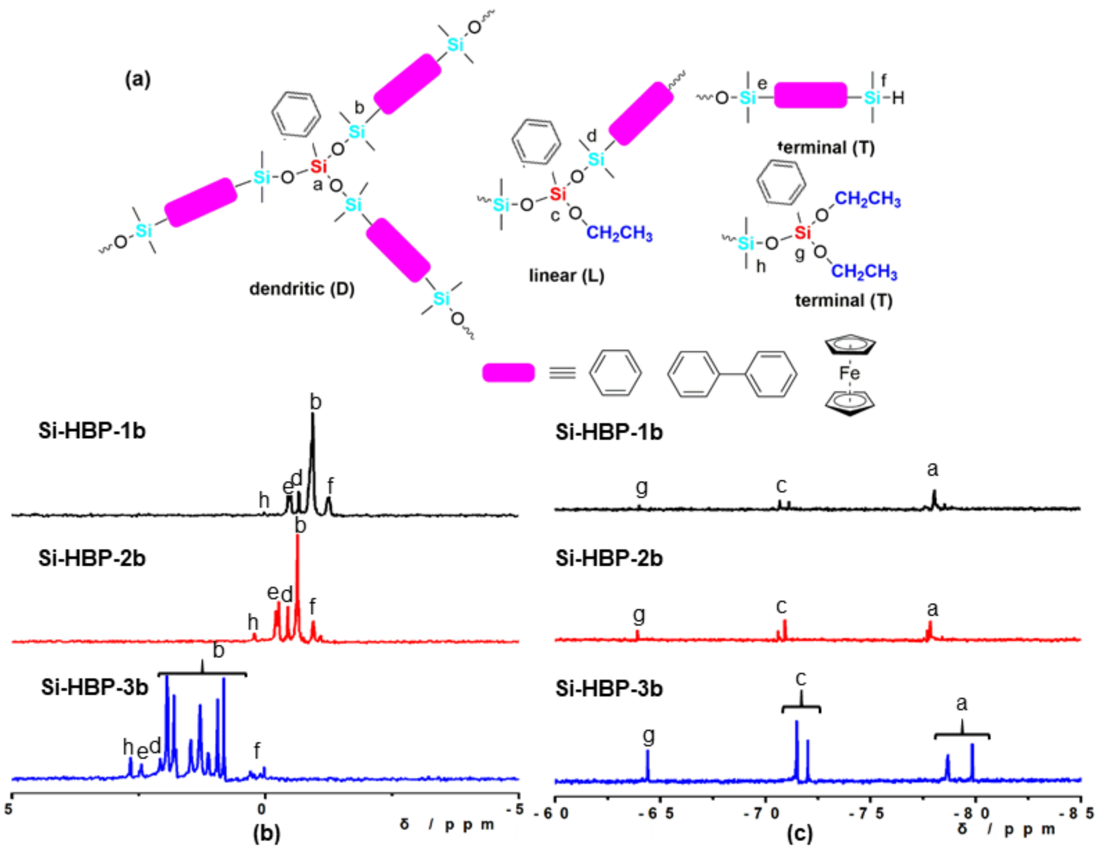

Figure S7. (a) The possible Si chemical environments for Si-HBPs; (b-c) ^{29}Si NMR spectra of Si-HBP-1b, Si-HBP-2b and Si-HBP-3b in the region of 5–-5 ppm and in the region of -60–-85 ppm.

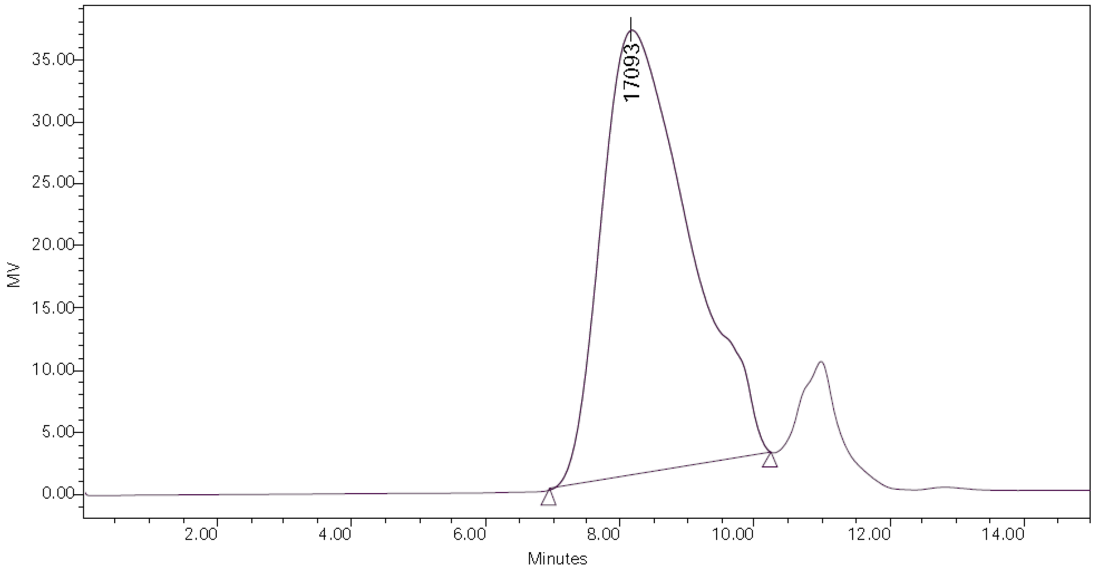

Figure S8. GPC curve of Si-HBP-1a.

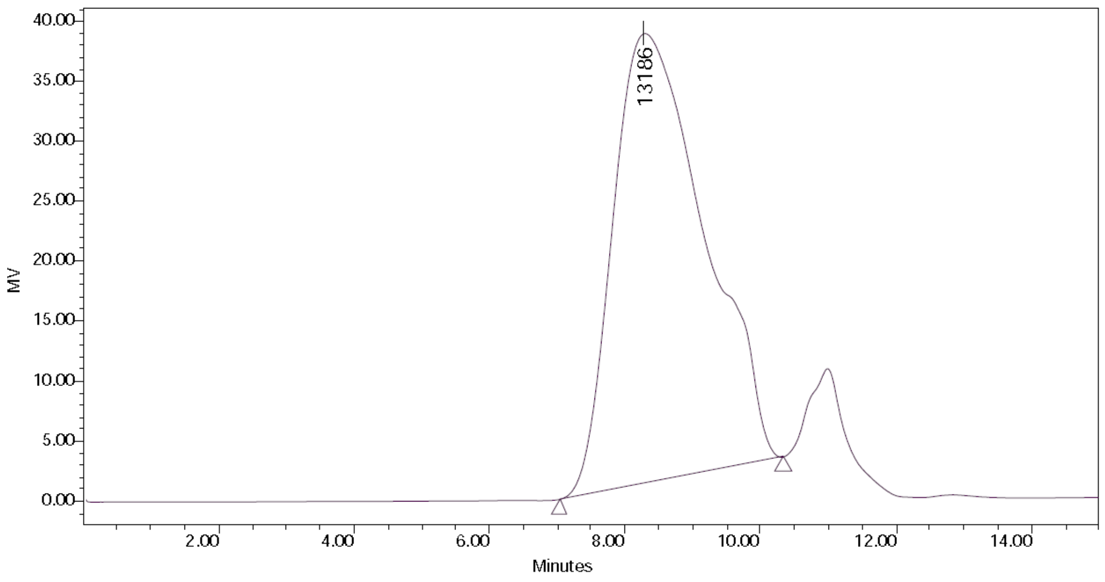

Figure S9. GPC curve of Si-HBP-1b.

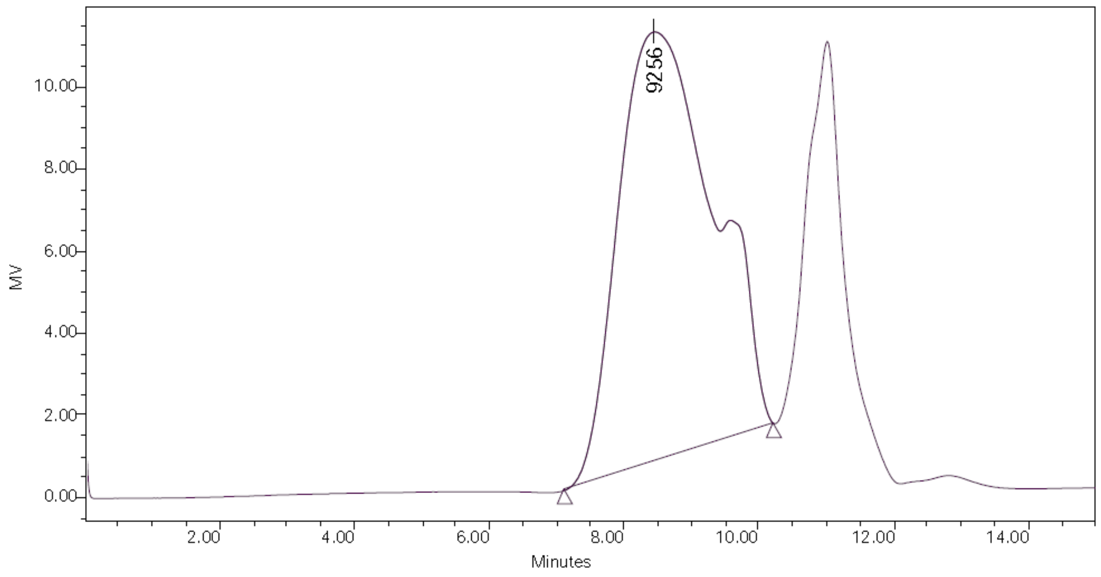

Figure S10. GPC curve of Si-HBP-2a.

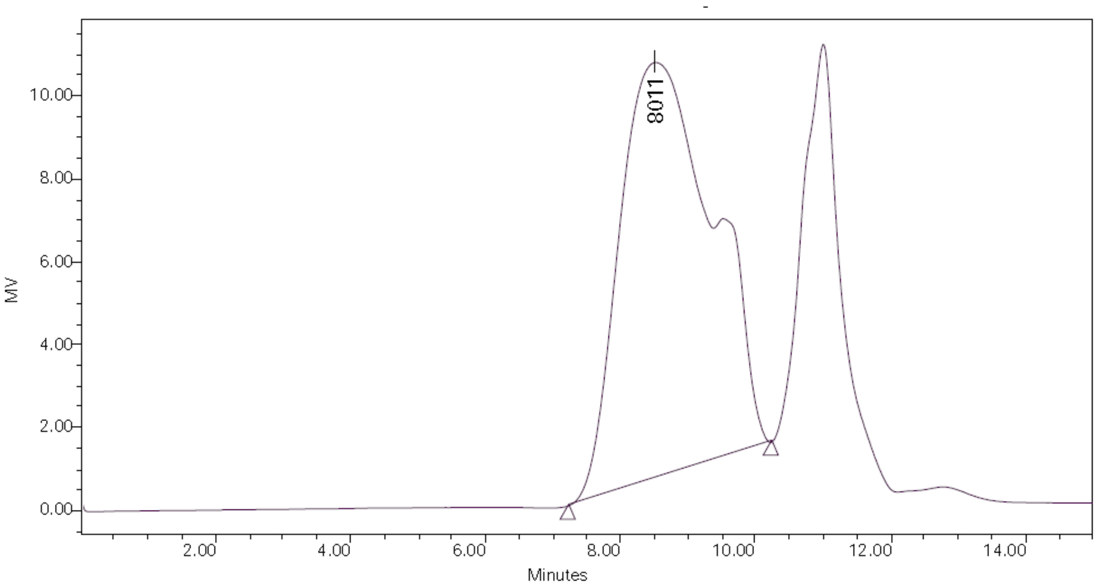

Figure S11. GPC curve of Si-HBP-2b.

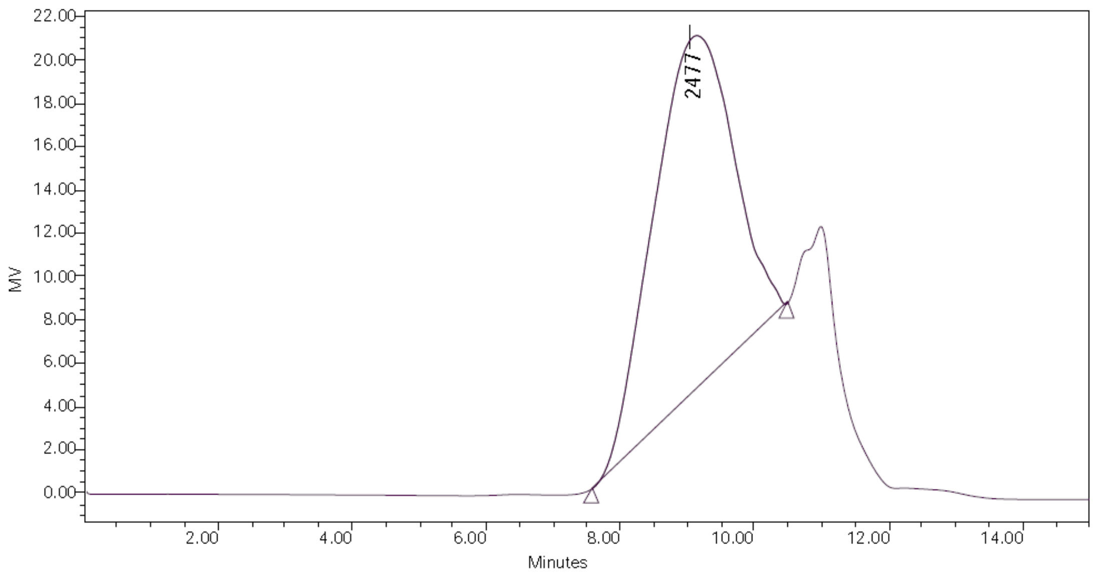

Figure S12. GPC curve of Si-HBP-3a.

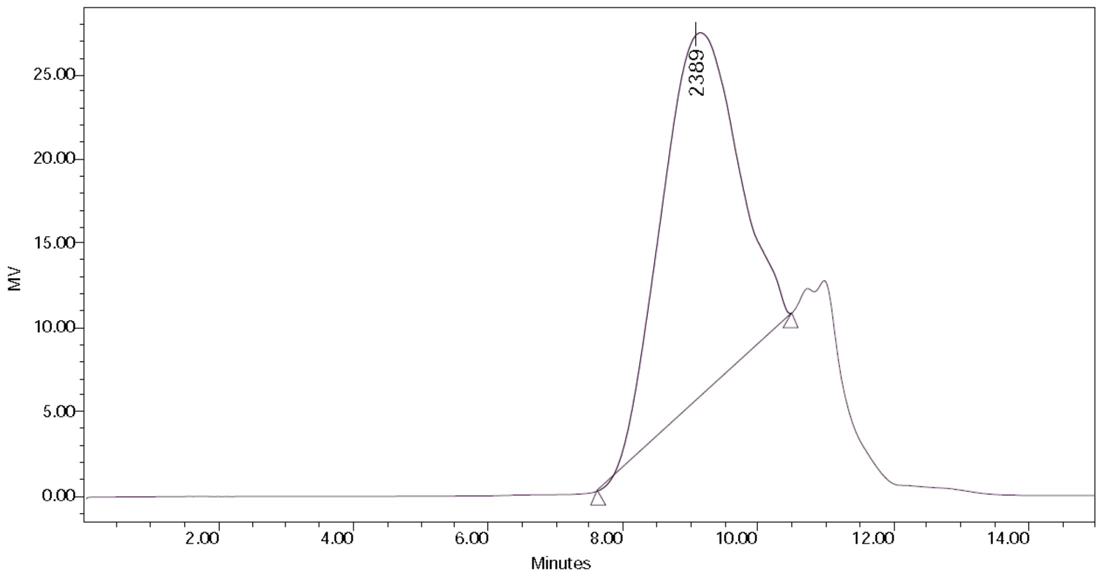

Figure S13. GPC curve of Si-HBP-3b.

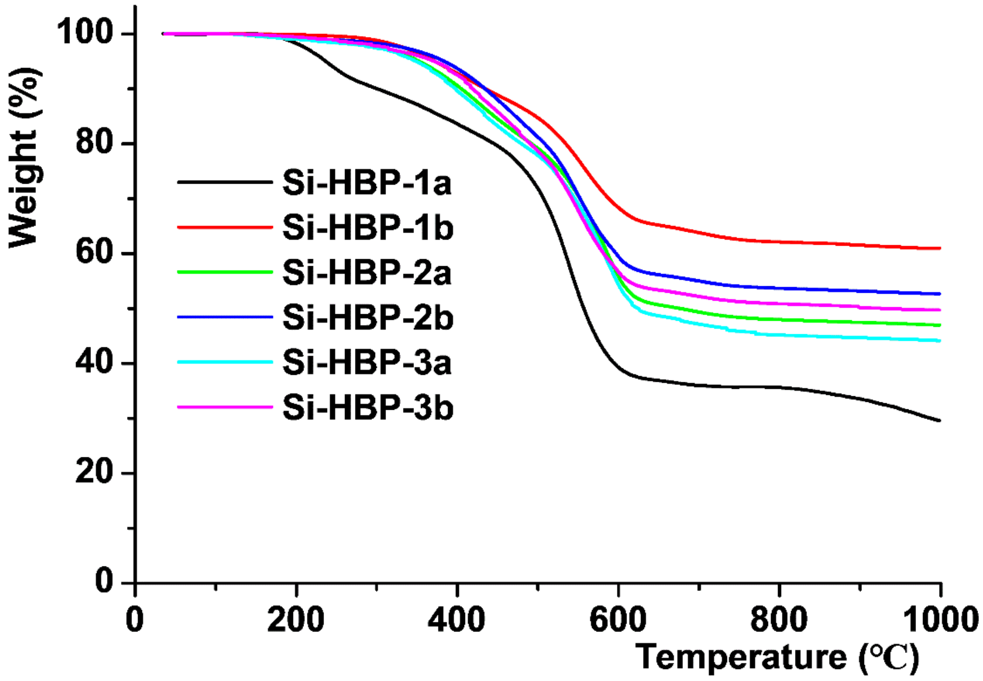

Figure S14. TGA curves of hyperbranched polycarbosiloxanes, Si-HBP-1~Si-HBP-3, under the atmosphere of nitrogen from temperature to 1000°C.
